# A Comparison of Emotional-Behavioral Problems of Siblings at the Age Range of 3-9 Year Old Children with Autism and Down Syndrome

**Published:** 2018

**Authors:** Nahid POURBAGHERI, Navid MIRZAKHANI, Alireza AKBARZADEHBAGHBAN

**Affiliations:** 1 Physiotherapy Research Centre, School of Rehabilitation, Shahid Beheshti University of Medical Sciences, Tehran, Iran.

**Keywords:** Siblings, Emotional behavioral problem, Autism, Down syndrome

## Abstract

**Objective:**

Children's emotional-behavioral problems will have a huge impact on their future. Such problems are more seen in the siblings of children with special needs. The present study aimed to compare emotional-behavioral problems in the healthy siblings of autistic children with the healthy siblings of children with Down syndrome in order to identify such children in Iran.

**Materials & Methods:**

This descriptive study was carried out in Tehran, Iran in 2016 on 174 healthy children aged between 3 and 9 yr old among whom 58 cases had autistic siblings, 58 cases had siblings with Down syndrome, and 58 cases had typically development siblings. The participants were selected using convenience sampling technique. All volunteer parents filled in SDQ Questionnaire*.* The study results were calculated using independent sample *t*-test, two-way ANOVA, and *Tukey* post hoc *test* by SPSS.

**Results:**

The mean overall score of Strengths and Difficulties Questionnaire was reported as 17.98±6.19 in the autism group, 11.01±6.56 in the Down syndrome group and 4.43±4.34 in the healthy group. There was a significant difference among autism, Down syndrome, and healthy groups. In the group of siblings with Down syndrome, the problems were significantly more in the age range of 3 to 7 yr old (*P*<0.05). However, no significant difference was observed in the scores of males and females (*P*>0.05).

**Conclusion:**

Siblings with autism or Down syndrome can have especial psychological effects on healthy children in families in the way that such effect will be more with autistic children. Therefore, formulating beneficial strategies for their parents is used to prevent emotional behavioral problems.

## Introduction

The prevalence of emotional-behavioral disorders in children is 1 per 5 cases. Children’s emotional-behavioral problems go under the rubric of two main classifications of externalizing and internalizing problems. Externalizing behavior problems refer to outside and include behaviors such as externalization, militancy, and disobedience. Within two disorders, hyperactivity and conduct disorder are diagnosed. On the other hand, internalizing problems refer more to inside and is observed as isolation, depression, and anxiety (1). Identification of such children is very important for mental health experts. One of the groups at risk is healthy siblings of children with disabilities (2, 3).

 The inability of one child not only influences parents but also brings about various issues for all members of the family. Communication within a family with a suffering child leads to a stressful communication and their healthy siblings have low self-esteem and self-confidence and are exposed to the risk of mental health due to the high expectations and reduction of their parents’ attention to them(4, 5). 

Among children with special needs, the birth of children with autism can be traumatic for the family because no disorder can affect other family members with autism and mental retardation (2). Autism is a type of pervasive developmental disorder associated with a severe injury in social interaction, communicational skills and stereotypical behaviors, interests, and activities (6). Down syndrome is also the most common genetic disorder known as the main cause of intellectual disabilities (7). 

In a bulk of studies, having siblings with special needs, especially autism and Down syndrome have been associated with negative effects. Such children experience less welfare than children with healthy siblings (8, 9) as well as an increase in emotional-behavioral problems (10). In the meantime, siblings of children with autism indicate more emotional-behavioral problems including aggression, depression and social isolation compared to siblings of children with Down syndrome (3, 11). Another group of studies reported the ineffectiveness or even positive effects of children with autism or Down syndrome on their healthy siblings (12, 13). In the studies on siblings of children with Down syndrome, the children had a good self-confidence and acted in dealing with problems in a more mature way (14, 15). Although autistic children may increase the risk of autism in other children of families, it would not increase behavioral-emotional problems in the healthy siblings and it should not be considered the cause of adjustment problems with other children (9). In addition, some studies reported positive and negative consequences of having siblings with Down syndrome or autism (11, 16). Researchers attribute the reason for the inconsistency on different results and effects of having disabled siblings on people to cultural and social factors, economic differences among families, age and sex of the children and the use of various assessment methods (17, 18) and in fact, the results obtained from such studies cannot be generalized to other countries. Despite the importance of mental health of siblings of children with autism and Down syndrome, this issue has been neglected about them (12).

We aimed to compare emotional-behavioral problems in the healthy siblings of children with autism and the healthy siblings of children with Down syndrome in order to know the mental space of the house and further investigate the effects of these disorders on Iranian families and healthy children.

## Materials & Methods

This descriptive study was carried out in Tehran, Iran in 2016 on 174 children in three groups with 58 cases consisted of healthy siblings aged between 3 and 9 yr old of children with autism, Down syndrome and healthy children. Tehran was first divided into five districts of East, West, Central, North, and South. Then, of each district, two rehabilitation centers were selected using convenience sampling technique. Then, the subjects were selected among the siblings of children with autism and Down syndrome referred to these 10 centers. However, no qualified one was found from the centers located in the north of the city. In addition, from the same region, 10 elementary boys and girls schools were selected using convenience sampling technique and 58 children with healthy siblings were selected as the healthy group. The inclusion criteria were the age range of 3 to 9 yr old, no history of mental and physical illness, having at least one brother or sister with autism or Down syndrome, passing at least 6 months from the disease of siblings with autism or Down syndrome, and obtaining a minimum score of 52 Gars-2 scale by children with autism to determine the extent and severity of autism. Moreover, the exclusion criterion was non-cooperation of the families. 

The data collection instruments were demographic questionnaire, Gars-2 scale, Strengths, and Difficulties Questionnaire (SDQ). Demographic questionnaire including questions related to the personal information of suffered children and their siblings was designed by the researcher himself. 

Gars-2 scale determines the probability and intensity of being autistic and can be used for people aged between 3 and 22 yr old. This test consists of three subscales of stereotypic behavior, communications, and social interaction. Each subscale consists of 14 items and each one is scored from zero to three. The scores of each subscale are summed and converted to standard scores. If a child's score is 52 or less, the child is less likely autistic. If a child's score is between 53 and 84, the probability of autistic child is medium. If a child's score is 85 or more, the child suffers from severe autism. This test has been normalized for people from 3 to 18 yr old in Iran. According to a study, Gars Scale was simultaneously used to estimate the validity of structure so that the correlation coefficients of two questionnaires was obtained 0.80 and the test cutoff point was 52. The reliability of the scale was estimated to be 0.89 using Cronbach's alpha coefficient too. The experience of positive prediction has proved the ability of Gars-2 to distinguish autistic persons from mentally retarded children, children with multiple disabilities, and children without disabilities (19).

The SDQ questionnaire evaluates the problems and abilities of children in the age range of 3 to 16 yr old using 25 items. Each question has been classified into the responses of right, somewhat right and wrong in which the reporter should choose one of them. The questionnaire consists of four similar self-report, teacher and parents forms in a way that in this study, the parent form containing two forms (children 3 to 4 yr) and (children 4 to 16 yr) was used. Indicators obtained from the questionnaire are reported as the overall scores of behavioral-emotional problems, emotional symptoms, conduct problems, hyperactivity, peer communications problems and prosocial behaviors. The diagnostic reliability and validity have been achieved at an acceptable level. SDQ total score is more sensitive in the diagnosis of problems in children and adolescent samples of the general population compared to Children Behavioral Check List (CBCL) and is better able to report hyperactivity of the samples (20). Through the implementation of parent and teacher form in Shahrekord, was obtained the reliability of parental form AS 0.42 using Cronbach's alpha coefficient and total cut-off point of 19 (21). This questionnaire has also been investigated in several other studies with similar results (22).After obtaining the permission and coordination with the management of selected rehabilitation centers, parents who had healthy children aged between 3 and 9 yr old in addition to children with autism or Down syndrome were first invited to the study. After they were familiarized with the study objectives, the consent form was distributed to them and the parents were asked to read it and complete it if they agreed with. Then, Gars-2 scale was only distributed among families of children with autism while demographic and SDQ questionnaires were distributed among families in the two groups. The questionnaires were filled out in the presence of a therapist so that he could orally explain any ambiguity in the items. After collecting all the questionnaires, children’s scores were calculated based on the scoring guidelines of SDQ and Gars-2 scale.

The ethical principles considered in the study included informing and obtaining informed consent, preserving the information in the questionnaires, excluding from the study in the case of unwillingness to contribute, failure to impose costs on families and lack of effect on the life process of the families. 

Homogeneity of the groups was evaluated in terms of age and gender taking into account 95% confidence level and 80% power using Chi-square test. In order to access significant difference between the groups in terms of the subscales of SDQ questionnaire, the effect of age was evaluated on the groups using two-way ANOVA. In the case of an interaction in each subscale, the analysis of subgroup including one-way ANOVA and Tukey post hoc test was used. Using independent t-test, two age groups of ( 3-7 yr=1 and 7-9 yr=2) were separately analyzed in each group and analyzed running SPSS Software V.21 (Chicago, IL, USA).

## Results

This study was conducted on three 58-member groups consisting of healthy 3 to 9 yr old siblings of children with autism, Down syndrome and healthy children. Gender distribution was determined as 37 boys and 21 girls in the autism group, 26 boys and 32 girls in the Down syndrome group, and 29 boys and 29 girls in the control group. The mean age of the three groups was 6.63±2.16, 6.77±2.09, and 6.2±1.99, respectively. In order to investigate the homogeneity of three groups in each mentioned variable, chi-square test was used so that the results showed no significant difference among the three groups in the mentioned variables and their counterpart result (*P*>0.05) (Table 1). 

**Table 1:** Group Means on Sibling emotional- behavioral problems, SDQ Scales

Variable	Siblings of children with DS M±SD		Siblings of children with ASD M±SD		Siblings of children with TD M±SD	
Age group (yr)	1	2	1	2	1	2
SDQ Subscale						
Emotional and behavioral problems	14.73±6.54	7.03±3.63	17.41±6.14	18.62±6.31	4.92±4.3	3.23±4.33
Emotional symptoms	3.26±2.01	1.96±1.31	4.03±2.57	3.92±2.33	.95±1.26	.52±1.32
Conduct problems	4.3±2.68	1.92±1.69	3.77±1.83	3.48±2.22	.65±.99	.35±.86
Hyperactivity/inattention	4.2±2.44	1.46±2.13	5.51±2.4	5.85±2.38	2.26±2.1	1.47±2.26
Peer problems	2.96±2.25	1.85±1.77	4.16±1.98	5.59±2.81	1.04±1.13	.88±1.36
Prosocial behavior	7.96±2.02	8.67±1.58	6.38±2.59	6.85±2.16	9.36±.94	9.00±1.32

ASD, autism spectrum disorder; DS, Down syndrome; TD, typically development; SD, standard deviation

The interaction effect of age was significant on the total scores of the questionnaire and subscales of conduct problems, hyperactivity, inattention, and peer problems (*P*<0.05) in which the comparison was done with age separation. Siblings aged between 3 and 7 yr old of children with autism and Down Syndrome Scale had significantly higher scores in the total scores of SDQ questionnaire and subscales of conduct problems, hyperactivity and inattention compared to the healthy children (*P*<0.001). However, there was no significant difference between autism and Down syndrome in the same age range (*P*>0.05) and that the siblings of children with autism significantly had more problems only in the subscale of problems with peers compared to the group with Down syndrome (*P*=0.02). In the age range of 7 to 9 yr old, the siblings of children with autism had significantly more problems compared to the siblings of children with Down syndrome and healthy groups in terms of the total score of the questionnaire (*P*=0.0001) and conduct problems (*P*=0.005), hyperactivity and inattention (*P*=0.0001), and problems with peers (*P*=0.0001). 

The siblings of children with Down syndrome had significantly more problems compared to the healthy group in terms of the total score of the questionnaire (*P*=0.03) and conduct problems (*P*=0.01) while there was no significant difference between problems with peers, hyperactivity, and inattention. The effect of age on both emotional symptoms and prosocial behaviors subscales was not significant. The comparison was done without taking into account the age factor. The siblings of children with autism had no significant difference with the siblings of children with Down syndrome in terms of emotional symptoms (*P*=0.0001), and prosocial behavior (*P*=0.001). On the other hand, both autism and Down syndrome groups had no significant different compared to the control group in terms of the above-mentioned subscales (*P*<0.05). In order to verify which age range had more problems in each group, the siblings aged between 3 and 7 yr old and 7 to 9 yr old were separately compared in each group. By comparing the two age ranges of 3 to 7 and 7 to 9 yr old in the Down syndrome group, behavioral-emotional problems (*P*=0.0001) of 3 to 7 yr old children with Down syndrome was significantly higher than that of 7 to 9 yr old children.

In addition, there was a significant difference among emotional problems (*P*=0.005), conduct problems (*P*=0.0001), inattention and hyperactivity (*P*=0.0001) and problems with peers (*P* =0.0001) while there was no significant difference in prosocial behavior scale. The 3 to 7-yr-old siblings of children with autism had only more problems compared to 7 to 9 yr siblings in terms of problems with peers (*P*=0.02) while there was no significant difference in the other subscales. The ethical principles considered in the study was ( IR.SBMU.RETECH.REC.1396.56)

## Discussion

The study compared emotional-behavioral problems in the siblings of children with autism with the siblings of children with Down's syndrome. Emotional-behavioral problems of the studied samples were 63.8% among the siblings of children with autism among which 13.8% were in the risk range. These numbers were reported 15.5% and 8.6% in the siblings of children with Down syndrome and 1.7% and 6.9% in the control group. Emotional-behavioral problems are more in the siblings of children with autism and Down syndrome than that in the healthy group such that in the age group of 7 to 9 yr old, problems were more in the autism group than in the Down syndrome group. These study results are consistent with a longitudinal study on the siblings of children with chronic disabilities that reported the siblings of children with autism experienced more adaptation problems (internal and external behavioral problems) compared to the other groups (3). 

In addition, autistic children's behavioral problems negatively affected the relationship with their siblings that predicted their depression and anxiety. Unlike the results obtained from this study, the siblings of children with Down syndrome lived well with their siblings (23), had good self-confidence, and acted more maturely in the face of problems (14, 15). The study on the siblings of children with autism was inconsistent with this study results. Although the presence of autistic children would not increase emotional-behavioral problems in the healthy siblings, it should not be considered the cause of adjustment problems to other children (9). This paradox is as a result of difference in how measurement, design, methodology, sample size, difference in the selected age range of the samples, number of data and group matching in terms of varying levels of intellectual disability (24). Factors such as gender, age, siblings’ household and caregiving responsibilities, and family variables could cause positive and negative outcomes in the behavioral problems of the siblings of children with autism and Down syndrome (7). 

In the current study, age significantly affected the siblings of children with Down syndrome and autism in a way that 3 to 7 yr siblings of children with Down syndrome significantly reported more problems for all SDQ subscales such as emotional problems, hyperactivity and inattention, conduct problems and peer problems compared to the control group while 7 to 9 yr siblings of children with Down syndrome significantly showed more problems only for conduct problems compared to the control group. Through comparing two age ranges of 3 to 7 and 7 to 9 yr old, we also concluded that emotional-behavioral problems are more in the 3 to 7 yr children with Down syndrome than that in the 7 to 9 yr children. Three to 7-yr-old siblings of autism reported more problems in terms of problems with peers compared to 7 to 9 yr siblings. There was no significant difference between the questionnaire scores of 3 to 7 yr children with autism and peers with siblings of children with Down syndrome in terms of the subscales while 7 to 9 yr old children with autism reported more problems in all subscales compared to 7 to 9 yr old children with Down syndrome. Thus, according to the samples under study, 3 to 7 yr old children are exposed to more problems. In line with the above-mentioned results, the younger children with autism or Down syndrome siblings showed more behavioral problems (15). In this regard, in a longitudinal study on Australian children, two time periods of 4 to 5 and 6 to 7 yr are sensitive periods for declining well-being in the healthy children of any family(1). In another longitudinal study, children in these two time periods received lower scores on SDQ questionnaire (8). 

Emotional-behavioral problems could stay in children up to adulthood which is the context of further problems. In this study, SDQ questionnaire was used to collect the required data. Accordingly, the age range of 4 to 5 yr old is one of the most important periods of growth in children and that children are more at risk for emotional- behavioral problems in this period. Unlike the results, a study was conducted on siblings of disabled children, birth order and the effects of their age gap. Older siblings of children with disabilities spent yr of their lives as normal, especially with lower age gap would be more susceptible to emotional-behavioral problems. In fact, the response of these children refers to the early years of their lives and they consider the presence of children with disabilities as a cause of anxiety and depression in their parents while the other younger siblings whose older siblings suffered from cognitive problems and disabilities at home experienced fewer emotional problems (25). 

In the present study, gender had no effect on creating emotional or behavioral disorders. In addition, these disorders were similar for both boys and girls. In this regard, the effect of gender was considered on emotional-behavioral problems neutral (26) while in several studies, in both autism and Down syndrome groups, boys showed behavioral problems as violence, aggression, poor social interaction with peers and feeling more competition with other siblings while girls indicated it as anxiety, depression and low self-confidence (25, 27, 28). The sisters of children with Down syndrome showed more behavioral and matching problems, especially in conduct problems compared to their brothers (14, 29-31) while in this study, both sisters and brothers of children with Down syndrome significantly received fewer scores compare to the siblings of children with autism and healthy groups in terms of conduct problems. The reason for this difference in the access to social protection, children’s level of information and knowledge about the problems related to their disabled siblings, unique characteristics of families, conditions, and type of dealing with problems and lifestyle (25, 31). 

One of the main results of this study was the positive and negative consequences in the samples under investigation. The siblings of children with autism significantly showed more prosocial behaviors such as respecting others’ feelings, sharing food and personal items with others, being kind to younger children and volunteering to help others. In line with the above-mentioned results, children with autism could cause stress for healthy children in family and that the experience of living in such conditions increases the risk of adjustment problems of the siblings of such children. However, despite the high vulnerability of emotional and behavioral disorders of some of the siblings, the presence of autistic children not only increases adjustment but also improves the exposure to adversity and disability. As a result, growing up with a sibling with autism can be both positive and negative for healthy siblings (17). Although the lower intellectual disability of children with Down syndrome, the higher stress levels of the family members and the higher the behavioral and emotional problems of the healthy children will be, the sibling of the child with Down syndrome can help their brother or sister to be stronger and more patient and act more maturely in dealing with other people with disabilities (7). 

In general, similar to our study, few studies compared the two groups of Down syndrome and autism and that most studies considered a wide range of intellectual disabilities. The samples limited to children at the age range of 3 to 9 yr old and convenience sampling technique due to the one-child family wishes and using the parent form of SDQ questionnaires and Gars-2 scale made the generalizability of the results very difficult. Therefore, along with investigating emotional-behavioral problems of the siblings, future research concentrate on other disabilities and in different age periods as well as the use of questionnaires which take into account the views of teachers and children themselves in addition to those of the parents. In addition, in this study, matching was merely done for age, gender, and severity of autism. Therefore, more confounding variables especially the economic status of families, social support and level of disability of all participants be controlled in future studies so that the studies provide the therapists and counselors of children with more comprehensive results. In this way, in planning for children with special needs, the family and healthy siblings are also taken into consideration with a wider view and training and support plans are formulated to maintain their mental health

**In Conclusion, **sibling emotional-behavioral condition in families of children with autism was characterized by more emotional-behavioral problems than those of the two comparison groups both siblings of children with autism and siblings of children with Down syndrome reported greater admiration of their sibling and less quarreling and competition in their relationships relative to normally developing comparison children. Siblings with autism or Down syndrome can have especial psychological effects on healthy children in families such that this effect will be more with autistic children. Despite problems such as depression, anxiety, hyperactivity, and difficulty in communicating with peers, having siblings with autism can probably increase prosocial behaviors.
